# Massive primary cardiac synovial sarcoma of the left atrium: a case report

**DOI:** 10.1186/s13019-022-01822-w

**Published:** 2022-04-14

**Authors:** Alice L. Zhou, Meghan E. Halub, John M. Gross, Benjamin L. Shou, Ahmet Kilic

**Affiliations:** 1 Division of Cardiac Surgery, Department of Surgery, Johns Hopkins Hospital, Zayed Tower, 1800 Orleans Street, Baltimore, MD 21287 USA; 2 Bone and Soft Tissue Surgical Pathology, Department of Pathology, Johns Hopkins Hospital, Baltimore, MD USA

**Keywords:** Cardiac tumor, Synovial sarcoma, Tumor resection, Atrial flutter, Case report

## Abstract

**Background:**

Synovial sarcomas are tumors typically located in the extremities and characterized by a t(X;18)(p11.2;q11.2) chromosomal translocation. With only around 100 cases reported in the literature, cardiac synovial sarcomas are extremely rare.

**Case presentation:**

We describe a case of a 59-year-old male who presented to his primary care physician with chest pain, palpitations, and dyspnea and was diagnosed with atrial flutter. Following atrial ablation, a transthoracic echocardiogram incidentally revealed a 5.5 × 5.0 cm heterogeneous mass. Further workup found a heterogeneous mass with mild fluorodeoxyglucose uptake that was abutting the left atrium, left ventricle, and left pulmonary veins. The tumor was resected and confirmed to be a monophasic synovial sarcoma with a *SS18-SSX* gene fusion. Four months post-operative, the patient had recovered well from surgery. He is currently undergoing concurrent radiation and chemotherapy.

**Conclusions:**

Due to the rarity of this tumor, guidelines on diagnosis and treatment come only from case reports. Our case describes a primary cardiac synovial sarcoma arising from the left atrium in the atrioventricular groove in which diagnosis of atrial flutter preceded detection of the mass.

## Background

Synovial sarcoma is a mesenchymal neoplasm that represents only 5–10% of soft tissue sarcomas and is characterized by a t(X;18) balanced translocation [1]. Synovial sarcomas typically occur in the deep soft tissue of the extremities, but there have been rare case reports of synovial sarcomas of the heart [2]. Primary cardiac synovial sarcomas (PCSS) are extremely rare, representing less than 5% of all primary cardiac sarcomas [3]. Patients typically present with dyspnea, chest pain, palpitations, and other nonspecific symptoms and are commonly treated with surgical resection, followed by adjuvant chemoradiation therapy [3, 4]. Given the rarity of these tumors, there are currently no established treatment guidelines. Here, we present the case of a PCSS arising from the left atrium in the atrioventricular groove in which the patient was first diagnosed with atrial flutter.

## Case report

The patient is a 59-year-old male with a past medical history of hypertension, mitral valve prolapse, mixed hyperlipidemia, and stage 3 chronic kidney disease. In September 2020, he presented to his primary care physician with symptoms of chest pain, palpitations, and dyspnea on exertion that had been waxing and waning for the past several months. A chest x-ray at the time noted cardiomegaly and a possible small left pleural effusion, but no other abnormalities. His electrocardiogram showed atrial flutter with 2:1 atrioventricular conduction. He was referred to Cardiology, and he underwent typical atrial flutter ablation. Transesophageal echocardiogram (TEE) performed prior to atrial flutter ablation visualized a mildly dilated left atrium with global hypokinesis of the left ventricle. No mass was detected at this time. Following the atrial flutter ablation, his symptoms improved.

**Fig. 1:**
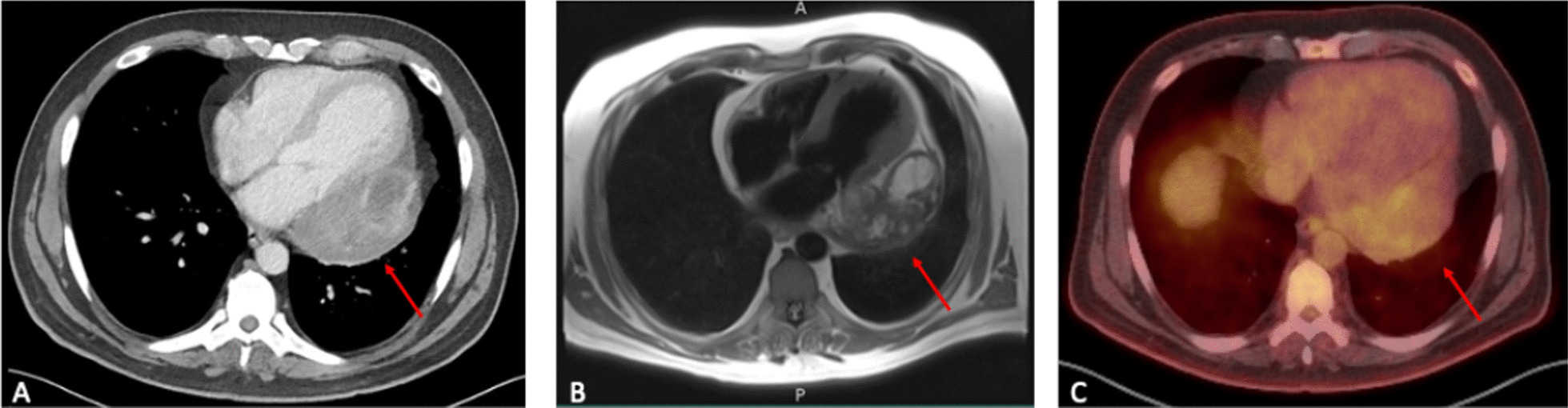
**A** CT scan showing a lesion along the left aspect of the pericardium. **B** MRI confirming the presence of a predominantly T1 isointense, mildly enhancing mass abutting the left atrium, left ventricle, and left pulmonary veins. **C** PET scan demonstrating a mass with mild heterogeneous FDG uptake

A routine follow-up transthoracic echocardiogram (TTE) from December 2020 revealed a 5.5 × 5.0 cm heterogeneous mass adjacent to the inferior/lateral wall of the left ventricle. The patient underwent further workup that included computed tomography (CT), magnetic resonance imaging (MRI), and positron emission tomography (PET) scans (Fig. 1). The lesion was a heterogeneous 9.8 × 5.7 × 7.0 cm cystic/soft tissue mass abutting the left atrium, left ventricle, and left pulmonary veins with peripheral rim enhancement along the anterior aspect and necrotic changes within. Mass effect on the left atrium was observed with preserved fat planes between the mass and the esophagus, and the circumflex artery was encased by the mass. The predominantly T1 isointense, mildly enhancing mass had a heterogeneous fluorodeoxyglucose (FDG) uptake with standard uptake value (SUV) max measuring up to 2.6.

The patient was referred to Cardiac Surgery for further workup and resection of the mass. Presurgical coronary angiography revealed that the left circumflex artery subtended two large collateral branches that supplied the vascular atrial mass. The patient underwent surgical resection of the mass through a median sternotomy utilizing cardiopulmonary bypass with central cannulation. The tumor was found to be intrapericardial, arising from and significantly compressing the left atrium in the atrioventricular groove and encompassing the entirety of the pericardium from the diaphragmatic surface to the pericardial reflection above the main pulmonary artery. Given the size of the tumor and the lack of a clear plane between the left ventricle and the mass, an aortic cross-clamp was placed and antegrade cardioplegia was utilized for myocardial protection. No cardiac chambers were opened as the mass was arising from the outer surface of the heart.

**Fig. 2:**
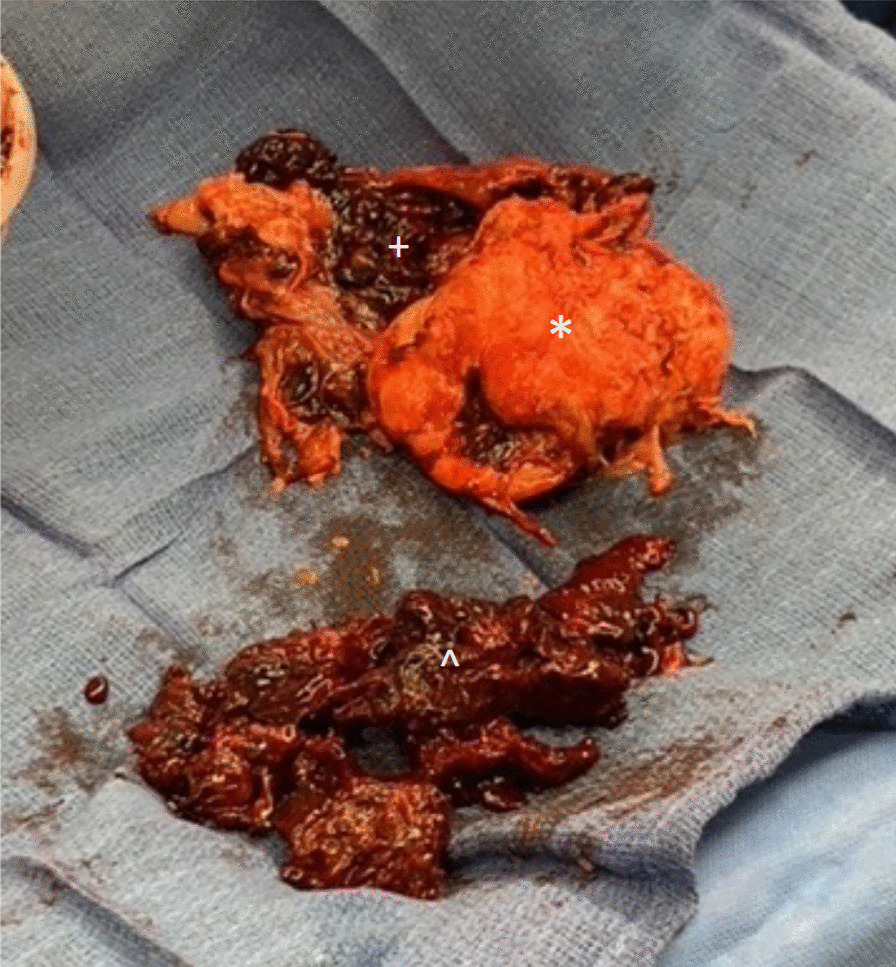
Surgical pathology. The mass (*) is a 10.0 × 8.2 × 4.5 cm cardiac synovial sarcoma with a surrounding capsule (+) and is surrounded by hemorrhage (^) for a large 22.0 × 12.0 × 6.0 cm hemorrhagic soft tissue mass that encompassed the entirety of the length of the mediastinum

The mass had encapsulated a portion of the second obtuse marginal (OM2) artery and was densely adhered to the left circumflex artery (LCX) and the left pulmonary veins. The tumor had a capsule surrounding it with a hemorrhagic component (Fig. 2). To resect as much of the tumor as possible, the LCX was shaved off and a small portion of the OM2 artery was resected along with the mass. The neoplasm vascularization was interrupted through cauterization during the dissection. Coronary artery bypass grafting (CABG) × 2 was subsequently performed using vein grafts to the LCX and the OM2. Reconstruction of the left superior pulmonary vein with a bovine pericardial patch was also performed. In the end, the bulk of the mass (> 98%) had been removed. The remaining tumor left behind was in the left atrioventricular groove, along the hilum of the left lung at the confluence of the pulmonary veins. Total cardiopulmonary bypass time was 320 min and total cross-clamp time was 195 min. Given the long cardiopulmonary bypass time and the significant amount of bleeding from the tumor bed, the patient needed to have coagulopathy corrected after resection, so the chest was left open. On post-operative day 2, the patient returned to the operating room for chest closure.

**Fig. 3:**
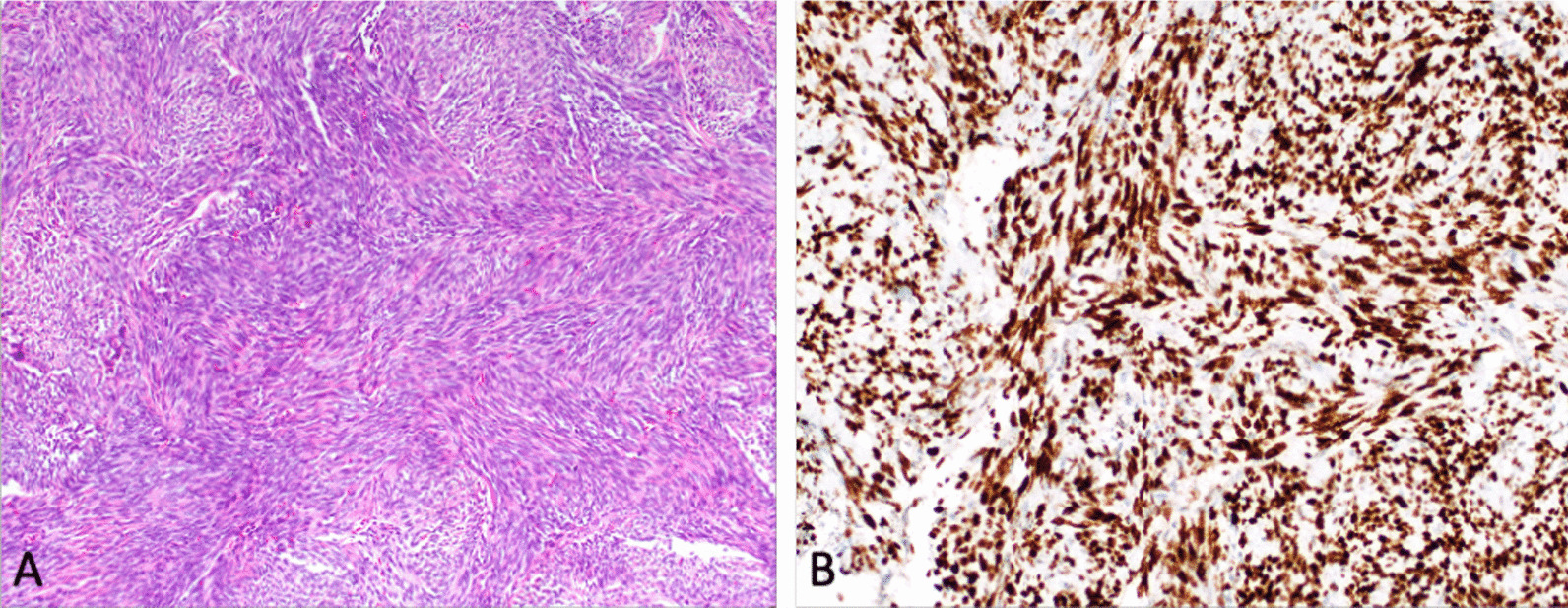
Pathology findings. **A** Hematoxylin and eosin stain at intermediate power (100 ×) showing classic morphology. **B** TLE1 immunohistochemistry showing strong and robust nuclear expression

The postoperative course was uneventful and the patient was discharged on post-operative day 10. Final pathology confirmed the diagnosis of a monophasic synovial sarcoma with a canonical *SS18-SSX1/SSX2* gene fusion. Immunohistochemistry (Fig. 3) found that the tumor was diffusely positive for TLE and negative for CK5/6, AE1/AE3, EMA, and S100. Four months later, the patient remained asymptomatic and had recovered well from surgery. He is currently undergoing concurrent radiation and chemotherapy with oral etoposide and oral cyclophosphamide.

## Discussion and conclusions

Synovial sarcomas are aggressive mesenchymal neoplasms typically located in the extremities and predominantly affecting young adults [1]. PCSS is extremely rare, representing less than 5% of all primary cardiac sarcomas [3]. To date, around 100 cases have been reported in the literature [4, 5]. PCSS has a 3:1 male predominance and a mean age of diagnosis in the fourth decade of life [3, 4]. These tumors are characterized by a t(X;18)(p11.2;q11.2) chromosomal translocation and the formation of a *SS18-SSX* fusion oncogene [4]. PCSS can be monophasic, which consists of only spindle cell components, or biphasic, which contains both spindle and epithelial cell components [6].

PCSS can originate from either the pericardium or the heart itself. Among intracardiac cases, the right side of the heart is involved in 71% of cases, with a predilection of arising from the atrium [4]. PCSS arising from the left atrium, as seen in our case, only occurs in 8.6% of cases [3]. Patients typically present with nonspecific symptoms, such as dyspnea, chest pain, and palpitations, due to mass effect [6]. Given its rarity, PCSS is difficult to diagnose. Chest x-rays frequently only detect secondary signs, such as cardiomegaly and pleural effusion [3], as seen in our case. CT scans, MRIs, and echocardiography can all detect the presence of the mass and define the tumor size but cannot distinguish PCSS from other soft-tissue neoplasms or masses [3]. Genetic analysis for detection of the *SS18-SSX* gene fusion must be used to confirm the diagnosis [2].

Given the rarity and heterogeneity of PCSS cases, there are no established treatment guidelines. Complete surgical resection has been associated with improved survival [4] but is often difficult, particularly when there is extensive involvement of the cardiac chambers, as seen with this patient. Adjunctive radiotherapy and chemotherapy are associated with greater survival, and the most commonly used chemotherapy regimen is ifosfamide and doxorubicin [3, 4]. Teng et al. [5] reported on the use of heart transplantation to treat a case of a PCSS invading the ventricular wall. However, it remains controversial whether transplantation prolongs survival in patients with cardiac sarcomas [7, 8].

Our case demonstrates a rare occurrence of a PCSS arising from the left atrium in the atrioventricular groove. In this case, a diagnosis of atrial flutter preceded detection of the mass. Due to the challenging anatomical location of the mass, an aggressive surgical approach was taken involving resection followed by bovine reconstruction and a two vessel CABG. Given the rarity of the disease, data on PCSS comes only from case reports. Our case adds to the existing literature, providing further insight into the diagnosis and treatment of PCSS.

## Data Availability

All data generated or analysed during this study are included in this published article [and its supplementary information files].

## Abbreviations

CABG: Coronary artery bypass grafting
CT: Computed tomography
FDG: Fluorodeoxyglucose
LCX: Left circumflex artery
MRI: Magnetic resonance imaging
OM2: Second obtuse marginal
PCSS: Primary cardiac synovial sarcoma
PET: Positron emission tomography
SUV: Standard uptake value
TTE: Transthoracic echocardiogram
